# Dependence of psychopharmacological drug effects on early social stress experience: An analysis aligned with the research domain criteria framework

**DOI:** 10.1016/j.jpet.2025.103710

**Published:** 2025-09-10

**Authors:** Margarita Moreno-Montoya, Elena Martín-González, Jeffrey W. Dalley

**Affiliations:** 1 Department of Psychology, Clinical & Experimental Neuroscience Research Group CTS280 and CIBIS (Centro de Investigación para el Bienestar y la Inclusión Social) Research Center, University of Almería, Almería, Spain; 2 Área de Psicobiología, Universitat Jaume I, Facultat de Ciencies de la Salut, Castellón de la Plana, Spain; 3 Department of Psychology, University of Cambridge, Cambridge, United Kingdom; 4 Department of Psychiatry, University of Cambridge, Cambridge, United Kingdom

**Keywords:** Stress, Repeated maternal separation, Research domain criteria, Environmental enrichment, Endocannabinoids, Monoamine neurotransmitters

## Abstract

Early life stress (ELS) refers to maltreatment, deprivation, and other traumatic events experienced early in life. This form of uncontrollable stress is widely acknowledged to cause long-term maladaptive changes in the developing nervous system, resulting in an increased risk for affective mood disorders with lasting impacts on mnemonic and other cognitive functions. ELS is often investigated in mice and rats by presenting stressors either during the perinatal period, repeatedly separating offspring from maternal care prior to the start of adolescence, or interventions involving maternal neglect. As discussed in this article, early social and environmental variables have profound and persistent effects on brain neurochemistry, which subsequently affect the pharmacological actions of psychoactive drugs, often in a sexually dimorphic manner. We first review the behavioral and neurochemical effects of ELS in rodents within the context of the research domain criteria framework, with a specific focus on different ELS protocols. We then discuss how ELS affects the pharmacological actions of psychoactive drugs that primarily target the brain monoaminergic, glutamatergic, and endocannabinoid systems. Our analysis demonstrates that ELS is a powerful modulator of neuropsychiatric drug action with implications for therapeutic efficacy in a range of affective and cognitive disorders in humans.

**Significance Statement:**

This review provides a comprehensive survey of salient research findings on the behavioral and neurochemical effects of early life stress (ELS) in experimental animals. Our analysis is informed with reference to the research domain criteria framework to evaluate the broader impacts of ELS on mental health. In addition, we discuss the effects of neuropharmacological agents to prevent or reverse the effects of ELS in adulthood.

## Graphical abstract

**Figure:**
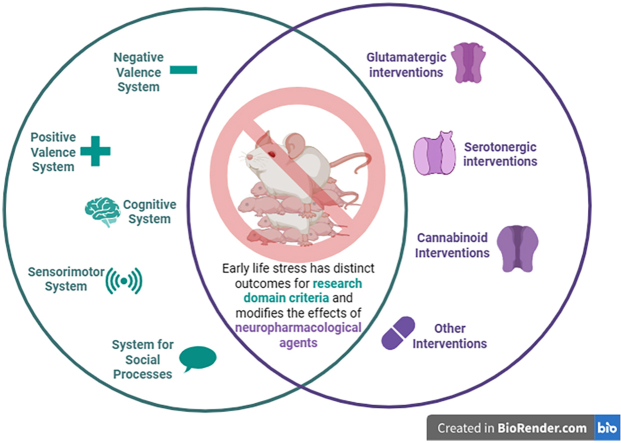

## 1 Introduction

Adverse experiences during early life encompass a wide range of uncontrollable events, including childhood maltreatment, neglect, parental separation, and various kinds of trauma.^1^, ^2^, ^3^ Early life stress (ELS) often manifests in a dysregulation of the hypothalamic-pituitary-adrenal axis, resulting in either hyperreactivity or hyporeactivity of the stress response system.^4^^,^^5^ Altered hypothalamic-pituitary-adrenal axis function is associated with increased vulnerability to psychopathology and impaired stress adaptation.^6^ Moreover, ELS has lasting effects on a myriad of neurotransmitter systems as well as brain structure and function, extending to the autonomic nervous system, immune processes, and circadian rhythms.^6^^,^^7^ These effects often persist throughout adulthood, increasing the risk for various disorders. The behavioral and neurobiological impacts of ELS include increased vulnerability to anxiety and depression, impaired social behavior, and cognitive impairment.^2^^,^^8^^,^^9^

Several rodent-based experimental approaches have been used to investigate the neurobehavioral consequences of ELS. These approaches aim to replicate various forms of adversity experienced early in life to investigate their long-term consequences on behavior, neurobiology, and overall health.^10^, ^11^, ^12^ As discussed further, the most widely used procedures in rodents include maternal separation (MS), limited bedding and nesting material (LBN), prenatal stress (PS), and early social stress.

### 1.1 MS

This widely used stress procedure involves separating rat or mouse pups from their mothers for extended periods during the early postnatal period.^13^ This intervention aims to simulate the stress of early maternal deprivation (MD), which can occur for various reasons in humans, including premature birth, hospitalization, and maternal illness. The age of separation is generally during the first 2 postnatal weeks, which coincides with a critical period for neurodevelopment in rodents.^14^, ^15^, ^16^, ^17^, ^18^ The control group typically involves unseparated pups that remain with their mothers, providing a baseline for comparison. However, despite its widespread use, the implementation of MS varies significantly in terms of the postnatal day when the intervention is implemented as well as the duration and frequency of each bout of separation.^13^

### 1.2 LBN material

This intervention focuses on manipulating the environment of the mother and pups by providing insufficient bedding material for building nests.^19^ This paradigm attempts to simulate the effects of neglectful caregiving often observed in human contexts where parents lack adequate resources or experience stress that impairs their ability to provide optimal care.^20^ In this procedure, the quantity of bedding material provided is significantly reduced, typically to a layer of roughly 1 cm. This restriction leads to disrupted maternal care as the mother struggles to maintain a comfortable and secure environment for her offspring. Mothers and pups in the control group are given abundant bedding material, typically 7–9 cm, thus allowing for normal nest building and maternal care.

### 1.3 PS

Although not strictly an ELS, PS involves exposing pregnant mothers to various stressors, such as restraint stress, forced swimming, or unpredictable noise.^21^ The purpose of these interventions is to investigate how maternal stress during pregnancy affects fetal development. PS procedures acknowledge that the prenatal environment can significantly influence offspring development and that maternal stress during pregnancy can have lasting effects on health and well-being. Various stressors can be used, including physical restraint, exposure to cold temperatures, and social stressors.^21^ The timing and duration of stress exposure during pregnancy can be varied to investigate sensitive periods of fetal development.

### 1.4 Early social stress

This model involves exposing young animals to social stressors, such as repeated brief isolation or interactions with aggressive conspecifics.^22^^,^^23^ This model seeks to simulate the effects of social adversity experienced in early life, such as bullying, social isolation, or exposure to violence. Different protocols use various social stressors, including repeated isolation, exposure to dominant individuals, or social instability. Like other early life stressors discussed earlier, social stress can also be applied during different developmental stages to investigate sensitive periods for social development.

The neurobehavioral effects of ELS are traditionally considered within a categorical framework without regard for transdiagnostic constructs that may more reliably facilitate translation between research in experimental animals and humans. We have thus adopted the principles of the research domain criteria (RDoC)^24^ approach to understand how ELS affects specific domains within the RDoC framework and so facilitate the identification of both new targets for treatment development and novel pathways to inform clinical decision making.^25^ This approach has led to the identification of psychologically significant phenotypes that may be studied from clinical and preclinical perspectives. However, to date, the application of the RDoC approach to preclinical research settings has been surprisingly limited.

By adopting the RDoC framework, the present review seeks to provide a clearer picture of the neurobehavioral effects of ELS from a transdiagnostic perspective. We specifically review how experimental approaches in rodents help us to understand the effects of ELS across the domains of negative valence, positive valence, social processes, cognitive systems, and sensorimotor systems. As a second related objective, we discuss how various neuropharmacological agents are affected by ELS procedures. Our analysis was informed by literature searches in PubMed and Web of Science, using a combination of keywords “early life stress,” “animal models,” “behavior,” and “neuropsychopharmacology” including the Boolean operators (and) for “cognition,” “memory,” “emotion,” “psychopharmacology,” and “treatments.” A total of 80 studies were included in the present review. Table 1^26^, ^27^, ^28^, ^29^, ^30^, ^31^, ^32^, ^33^, ^34^, ^35^, ^36^, ^37^, ^38^, ^39^, ^40^, ^41^, ^42^, ^43^, ^44^, ^45^, ^46^, ^47^, ^48^, ^49^, ^50^, ^51^, ^52^, ^53^, ^54^, ^55^, ^56^, ^57^, ^58^, ^59^, ^60^, ^61^, ^62^, ^63^, ^64^, ^65^, ^66^, ^67^, ^68^, ^69^, ^70^, ^71^, ^72^, ^73^, ^74^, ^75^, ^76^, ^77^, ^78^, ^79^, ^80^, ^81^, ^82^, ^83^, ^84^, ^85^, ^86^ summarizes the main findings of these studies according to the behavioral system and domains affected by ELS.

**Table 1:** Behavioral effects of early life stress in accordance to Research Domain Criteria for neuropsychiatric disorders

ELS Model	ELS Protocol	Subjects	Construct	Outcome Measure	Behavior	Citation
Domain: negative valence system						
MS	Daily, 3 h from PND1 to PND10	Male Wistar rats	Acute threat	FCT	↑ freezing in the nonconditioned context compared with animals that received handling but not compared with control group	Couto-Pereira et al,^26^ 2019
MS	Daily, 3 h from PND1 to PND10	Male and female Wistar rats	Acute threat	FCT	↑ freezing in both sexes	Diehl et al,^27^ 2014
MS	Daily, 15 min (brief MS group) or 3 h (long MS group) from PND2 to PND14	Male Wistar rats	Acute threat	FCT	↓ freezing	Guijarro et al,^28^ 2007
MS	Daily, 15 min (brief MS group) or 3 h (prolonged MS group) from PND1 to PND21	Male and female Sprague–Dawley rats	Acute threat	FCT	BMS ↓ decreased ultrasonic vocalization during the acquisition PMS ↓ ultrasonic vocalization in females	Kosten et al,^29^ 2006
LBN	From PND4 to PND11	Male and female C57BL/6N mice	Acute threat	FCT	LBN female mice ↓ freezing Higher intensity footshock was required to elicit an audible vocalization in LBN males and females LBN female mice required a higher intensity shock to vocalize during the estrus phase	Manzano-Nieves et al,^30^ 2018
MS	Daily, 6 h from PND1 to PND21	Male and female Wistar rats	Acute threat	FCT	MS female ↓ freezing	Sun et al,^31^ 2014
MS	Daily, 3 h from PND2 to PND15	Male and female BALB/cJ mice	Acute threat	FCT	MS mice ↓ freezing	Wang et al,^32^ 2011
MS	Daily, 3 h from PND2 to PND14	Female Sprague–Dawley rats	Acute threat	FCT	No differences during the postshock period and the recall of contextual conditioned fear 24 h later ↑ freezing on the last extinction day in MS female rats compared with control group	Xiong et al,^33^ 2014
MS	Daily, 3 h from PND1 to PND14	Male CD1 mice	Acute threat	FCT	=	Zoicas and Neumann,^34^ 2016
MS	Daily, 3 h from PND1 to PND10	Male and female Wistar rats	Acute threat	FCT	=	Andressa Caetano et al,^35^ 2024
MS	Daily, 3 h from PND1 to PND14	Male and female Wistar rats	Acute threat	FCT	MS male and female adolescent rats displayed ↓ freezing during day 2. In adulthood, MS (conditioned during adolescence) displayed ↓ freezing.	Chocyk et al,^36^ 2014
MS	Daily, 4 h from PND1 to PND10 (MS10) or from PND1 to PND21 (MS21)	Male Wistar rats	Acute threat	PAT	MS10 ↓ latency of crossing to the dark chamber during the recall phase	Banqueri et al,^37^ 2017
MS	Daily, 3 h from PND1 to PND14	Male Holtzman rats	Acute threat	Shuttle box escape test	=	Ali and Dwivedi,^38^ 2025
MS	Daily, 4.5 h from PND1 to PND21	Male and female Wistar rats	Acute threat	Step-down inhibitory avoidance task	↓ latency to step-down and ↓ freezing behavior	Vivinetto et al,^39^ 2013
MS	Daily, 3 h from PND2 to PND21	Males Wistar rats	Potential threat	EPM	↓ in the open arms	Aisa et al,^40^ 2007
MS	Daily, 3 h from PND2 to PND14	Female Sprague–Dawley rats	Potential threat	EPM	=	Xiong et al,^33^ 2014
MS	Daily, 3 h from PND1 to PND21	Male Sprague–Dawley rats	Potential threat	EPM	↑ in the open arms	Zhang et al,^41^ 2014
MS	Daily, 3 h from PND1 to PND14	Male CD1 mice	Potential threat	EPM	=	Zoicas and Neumann,^34^ 2016
MS	Daily, 3 h from PND2 to PND21	Male Sprague–Dawley rats	Potential threat	EPM	↓ center entry times, open arms time and open/closed arms time	Cao et al,^42^ 2016
MS	Daily, 3 h from PND1 to PND14	Male Holtzman rats	Potential threat	EPM	=	Ali and Dwivedi,^38^ 2025
MS	Daily, 3 h from PND2 to PND21	Male and female Sprague–Dawley rats	Potential threat	EPM	↑ time in the open arms	Kim et al,^43^ 2024
MS	Daily, 3 h from PND1 to PND10	Male and female Wistar rats	Potential threat	EPM	=	Andressa Caetano et al,^35^ 2024
MS	Daily, 3 h from PND2 to PND21	Male Sprague–Dawley rats	Potential threat	EPM	↓ time in open arms	Liang et al,^44^ 2024
MS	Daily, 3 h from PND1 to PND10	Male and female Wistar rats	Potential threat	EPM	MS males (but not females) ↑ time in open arms	dos Santos et al,^45^ 2023
MS	Daily, 6 h from PND4 to PND14	Male Wistar rats	Potential threat	Light/dark test	↑ time and ↓ latency to enter into the dark chamber	Kambali et al,^46^ 2019
MS	Daily, 3 h from PND2 to PND14	Male Wistar rats	Potential threat	Light/dark test	↓ time spent in the light box	Rincel et al,^47^ 2019
LBN	From PND2 to PND9	Male C57BL/6J mice	Potential threat	OFT	=	Rice et al,^48^ 2008
MS	Daily, 3 h from PND2 to PND21	Male Sprague–Dawley rats	Potential threat	OFT	↓ time in the center	Cao et al,^42^ 2016
MS	Daily, 3 h from PND2 to PND21	Male and female Sprague–Dawley rats	Potential threat	OFT	↑ time in the center	Kim et al,^43^ 2024
MS	Daily, 3 h from PND2 to PND21	Male Sprague–Dawley rats	Potential threat	OFT	↓ time in the center	Liang et al,^44^ 2024
MS	Daily, 3 h from PND1 to PND10	Male and female Wistar rats	Potential threat	OFT	MS females (but not males) ↑ time in the center	dos Santos et al,^45^ 2023
MS	Daily, 3 h from PND2 to PND21	Male and female Sprague–Dawley rats	Potential threat	OFT	MS group ↑ time in the center, and ↓ time in the edge	Kim et al,^49^ 2020
MD	24 h from PND9 to PND10	Male and female Wistar rats	Potential threat	OFT	↓ time in center	Rentesi et al,^50^ 2010
MS	Daily, 3 h from PND2 to PND21	Female Wistar rats	Loss	FST	↑ immobility in the second day of the test	Aisa et al,^51^ 2008
MS	Daily, 3 h from PND2 to PND21	Males Wistar rats	Loss	FST	↑ immobility in the second day of the test	Aisa et al,^40^ 2007
LBN	From PND2 to PND21	Male Sprague–Dawley rats	Loss	FST	↑ immobility	Cui et al,^52^ 2006
MS	Daily, 3 h from PND2 to PND12	Female Sprague–Dawley rats	Loss	FST	=	Pusceddu et al,^53^ 2015
MS	Daily, 3 h from PND2 to PND21	Male Wistar rats	Loss	FST	↑ immobility	Solas et al,^54^ 2010
MS	Daily, 3 h from PND2 to PND15	Male and female BALB/cJ mice	Loss	FST	=	Wang et al,^32^ 2011
MS	Daily, 3 h from PND1 to PND10	Male and female Wistar rats	Loss	FST	↑ immobility in female (during adolescence) and male (during adulthood)	Borba et al,^55^ 2021
MD	Daily, 3 h from PND1 to PND10	Male and female Wistar rats	Loss	FST	MS male (but not female) ↑ immobility and ↓ climbing	Abelaira et al,^56^ 2021
MD	Daily, 3 h from PND1 to PND10	Male Wistar rats	Loss	FST	↓ swimming at PND20 and ↓ immobility and ↓ climbing at PND60	Réus et al,^57^ 2017
MS	Daily, 3 h from PND1 to PND14	Male Holtzman rats	Loss	FST	=	Ali and Dwivedi,^38^ 2025
MS	Daily, 3 h from PND2 to PND21	Male and female Sprague–Dawley rats	Loss	FST	↑ immobility	Kim et al,^43^ 2024
MD	Daily, 3 h from PND1 to PND10	Male Wistar rats	Loss	FST	↑ immobility	Bertollo et al,^58^ 2024
MS	Daily, 6 h from PND2 to PND14	Male Wistar rats	Loss	FST	=	Montes-Rodríguez et al,^59^ 2024
MS	Daily, 3 h from PND2 to PND21	Male and female Sprague–Dawley rats	Loss	FST	↑ immobility and ↓ climbing	Kim et al,^49^ 2020
MS	Daily, 3 h from PND2 to PND21	Sprague–Dawley rats	Loss	FST	↑ immobility time	Gondré-Lewis et al,^60^ 2016
MS	Daily, 3 h from PND2 to PND21	Male and female Sprague–Dawley rats	Loss	TST	↑ immobility	Kim et al,^43^ 2024
MS	Daily, 3 h from PND2 to PND21	Male Wistar rats	Loss	SPT	↓ sucrose consumption	Aisa et al,^40^ 2007
MS	Daily, 3 h from PND1 to PND14	Male Holtzman rats	Loss	SPT	↓ sucrose preference	Ali and Dwivedi,^38^ 2025
MS	Daily, 3 h from PND2 to PND21	Male and female Sprague–Dawley rats	Loss	SPT	↓ sucrose preference	Kim et al,^43^ 2024
MS	Daily, 3 h from PND1 to PND14	Male and female Long Evans rats	Loss	SPT	=	Wilkinson et al,^61^ 2024
MS	Daily, 3 h from PND1 to PND10	Male and female Wistar rats	Loss	SPT	=	dos Santos et al,^45^ 2023
MS	Daily, 3 h from PND2 to PND21	Sprague–Dawley rats	Loss	SPT	↓ sucrose preference	Gondré-Lewis et al,^60^ 2016
MS	Daily, 3 h from PND2 to PND14	Male Wistar rats	Loss	SPT	↓ sucrose preference	Rincel et al,^47^ 2019
MS	Daily, 3 h from PND2 to PND14	Male Wistar rats	Loss	FUST	No preference for female urine	Rincel et al,^47^ 2019
LBN	From PND2 to PND9	Male rats	Loss	Voluntary access to highly palatable food test	↓ voluntary chocolate intake	Bolton et al,^62^ 2018
Domain: positive valence system						
MD	Daily, 3 h from PND1 to PND14	Male Long Evans rats	Reward learning	ASST	MD rats required more trials to obtain criterion for 3 stages of discrimination: compound discrimination, reversal 1 and extradimentional shift	Baudin et al,^63^ 2012
MS	Daily, 3 h from PND2 to PND21	Male Sprague–Dawley rats	Reward learning	rGT	MS group showed a smaller proportion of good decision makers and higher proportion of poor decision makers	Cao et al,^42^ 2016
ESI	Daily, 30 min from PND14 to PND21	Male and female Marchigian Sardinian and Wistar rats	Reward learning and reward responsiveness	Alcohol self-administration	=	Benvenuti et al,^64^ 2024
MS	Daily, 3 h from PND2 to PND21	Sprague–Dawley rats	Reward learning and reward responsiveness	Alcohol self-administration	↑ alcohol consumption	Gondré-Lewis et al,^60^ 2016
LBN	From PND2 to PND9	Male rats	Reward learning and reward responsiveness	Cocaine self-administration	LBN rats acquired cocaine SA faster than controls.	Bolton et al,^62^ 2018
LBN	From PND2 to PND9	Male Sprague–Dawley rats	Reward learning and reward responsiveness	Heroin self-administration	↓ heroin total infusions, ↓ heroin infusions during loading phase, and ↓ hedonic setpoint for opioids	Levis et al,^65^ 2022
MS	Daily, 3 h (MS180) or 15 min (MS15) from PND2 to PND14	Male Long Evans rats	Reward learning and reward responsiveness	Methamphetamine self-administration	MS180 ↑ active lever presses and ↑ infusions than MS15	Lewis et al,^66^ 2013
Wet bedding and nesting	PND5	Male and female Long Evans rats	Reward valuation	Conditioned suppression task	↑ latencies to collect reward in the presence of the fear-associated CS+ and ↑ suppression	Bercum et al,^67^ 2023
ESS	3 mo	Male Long Evans rats	Reward valuation (delay)	Delay-to-reinforcement task	↑ preference of the delayed lever compared with enriched rats	Hellemans et al,^68^ 2005
ESS	During the whole experiment	Male Lister Hooded rats	Reward valuation (delay)	Temporal discounting of reward task	↑ preference for the delayed, larger reward	Liu et al,^69^ 2017
ESI	Daily, 30 min from PND14 to PND21	Male and female Marchigian Sardinian and Wistar rats	Reward valuation (effort)	PRSR alcohol self-administration	=	Benvenuti et al,^64^ 2024
Domain: cognitive system						
MS	Daily, 3 h from PND1 to PND14	Male and female Wistar rats	Cognitive control	5CSRTT	↓ omissions during the Basle Line, the SITI, and the LITI challenge. ↑ perseverative responses during the Base Line and the SD0,5 challenge	Boutros et al,^64^ 2017
IR	During the whole experiment	Male Sprague–Dawley rats	Cognitive control	5CSRTT	↓ premature responses	Tung et al,^70^ 2023
IR	During the whole experiment	Male Lister hooded rats	Cognitive control	5CSRTT	tendency ↑ perseverative nose pokes during the acquisition stage	Liu et al,^69^ 2017
MS	Daily, 6 h from PND4 to PND14	Male Wistar rats	Cognitive control	5CSRTT	similar	Kambali et al,^46^ 2019
MS	Daily, 3 h from PND2 to PND21	Male and female Sprague–Dawley rats	Cognitive control	CAT	↑ time in Borden and floor zone.	Kim et al,^43^ 2024
MS	Daily, 3 h from PND2 to PND21	Male and female Sprague–Dawley rats	Cognitive control	VCAT	↓ jumping latency	Kim et al,^43^ 2024
MS	Daily, 3 h from PND1 to PND21	Male Sprague–Dawley rats	Spatial memory	MWM	=	Zhang et al,^41^ 2014
LBN	From PND2 to PND9	Male Sprague–Dawley rats	Spatial memory	MWM	LBN failed to reduce their platform-finding time on successive trials	Brunson et al,^71^ 2005
LBN	From PND2 to PND21	Male Sprague–Dawley rats	Spatial memory	MWM	↑ latencies to escape onto the hidden platform	Cui et al,^52^ 2006
MS	Daily, 3 h from PND2 to PND14	Male Sprague–Dawley rats	Spatial memory	MWM	↑ latencies to find the platform	Dallé et al,^72^ 2017
LBN	From PND2 to PND9	Male and female C57B1/6J mice	Spatial memory	MWM	The latency to find the platform decreased between the first and the last training day in all groups except for the LBN males	Naninck et al,^73^ 2015
LBN	From PND2 to PND9	Male C57BL/6J mice	Spatial memory	MWM	↑ latencies to find the platform	Rice et al,^48^ 2008
MS	Daily, 3 h from PND2 to PND21	Male Wistar rats	Spatial memory	MWM	=	Solas et al,^54^ 2010
MS	Daily, 6 h from PND1 to PND21	Male and female Wistar rats	Spatial memory	MWM	=	Sun et al,^31^ 2014
MS	Daily, 4 h from PND1 to PND 21 (MS21 group) or from PND1 to PND10 (MS10 group)	Female Wistar rats	Spatial memory	MWM	=	Banqueri et al,^74^ 2018
MS	Daily,1 h from PND2 to PND9	Male Sprague–Dawley rats	Spatial memory	MWM	↑ latencies to find the platform at day 1 of training	Huang et al,^75^ 2002
LBN	From PND2 to PND9	Male Sprague–Dawley rats	Spatial memory	MWM	↑ latencies to find the platform	Ivy et al,^76^ 2010
LBN	From PND2 to PND9	Male 129S2/Sv X C57BL/6J mice	Spatial memory	MWM	↑ latencies to find the platform	Wang et al,^32^ 2011
MS	Daily, 3 h from PND2 to PND14	Male and female Sprague–Dawley rats	Spatial memory	MWM	↑ latencies to find the platform	Xu et al,^77^ 2018
MS	Daily, 3 h from PND1 to PND21	Male Wistar rats	Spatial memory	MWM	=	Xue et al,^78^ 2013
MS	Daily, 6 h from PND4 to PND14	Male Wistar rats	Spatial memory	RAM	↑ % correct choice during spatial learning = % correct choice during remote spatial memory test	Kambali et al,^46^ 2019
LBN	From PND2 to PND9	Male C57BI6, C57BL/6N mice	Spatial memory	OLT	↓ discrimination index	Kanatsou et al,^79^ 2017
LBN	From PND2 to PND9	Male and female C57B1/6J mice	Spatial memory	OLT	↓ ratio novel/familiar object location exploration	Naninck et al,^73^ 2015
LBN	From PND2 to PND9	Male C57B1/6J Mice	Spatial Memory	OLT	↓ ratio novel/familiar object location exploration.	Naninck et al,^80^ 2017
MD	101 Daily, 3 h from PND1 to PND14	Male and female Long Evans rats	Spatial memory	OLT	Lower performance	Mourlon et al,^81^ 2010
LBN	From PND2 to PND9	Male C57B1/6J Mice	Spatial memory	OLT	↓ discrimination of the novel location	Hoeijmakers et al,^82^ 2018
MS	Daily, 3 h from PND2 to PND21	Female Wistar rats	Nonspatial Memory	NOR	↓ discrimination index	Aisa et al,^51^ 2008
LBN	From PND2 to PND9	Male Sprague–Dawley rats	Nonspatial memory	NOR	↓ discrimination of the novel object	Ivy et al,^76^ 2010
LBN	From PND2 to PND9	Male Sprague–Dawley rats	Nonspatial memory	NOR	↓ discrimination of the novel object	Molet et al,^83^ 2016
LBN	From PND2 to PND9	Male and female C57B1/6J mice	Nonspatial memory	NOR	↓ discrimination of the novel object	Naninck et al,^73^ 2015
MS	Daily, 3 h from PND2 to PND12	Female Sprague–Dawley rats	Nonspatial memory	NOR	=	Pusceddu et al,^53^ 2015
MS	Daily, 3 h from PND2 to PND14	Male C57B1/6 mice	Nonspatial memory	NOR	↓ discrimination of the novel object	Reshetnikov et al,^84^ 2018
LBN	From PND2 to PND9	Male C57BL/6J mice	Nonspatial memory	NOR	↓ discrimination of the novel object	Rice et al,^48^ 2008
MS	Daily, 3 h from PND2 to PND21	Male Wistar rats	Nonspatial memory	NOR	↓ discrimination of the novel object	Solas et al,^54^ 2010
MS	Daily, 3 h from PND2 to PND15	Male and female BALB/cJ mice	Nonspatial memory	NOR	↓ discrimination of the novel object	Wang et al,^32^ 2011
MS	Daily, 3 h from PND1 to PND14	Male CD1 mice	Nonspatial memory	NOR	=	Zoicas and Neumann,^34^ 2016
MS	Daily, 4.5 h from PND1 to PND21	Male and female Wistar rats	Nonspatial memory	NOR	=	Vivinetto et al,^39^ 2013
MS	Daily, 3 h from PND2 to PND15	Female Balb/c mice	Nonspatial memory	NOR	↓ discrimination of the novel object	Wearick-Silva et al,^85^ 2017
MD	Daily, 3 h from PND1 to PND14	Male and female Long Evans rats	Nonspatial memory	NOR	=	Mourlon et al,^81^ 2010
MS	Daily, 4 h from PND1 to PND10 (MS10) or from PND1 to PND21 (MS21)	Male Wistar rats	Nonspatial memory	NOR	MS 10 and MS21 ↓ discrimination of the novel object during adolescence MS21 ↓ discrimination of the novel object during adulthood	Banqueri et al,^37^ 2017
MS	Daily, 4 h from PND1 to PND 21 (MS21 group) or from PND1 to PND10 (MS10 group)	Female Wistar rats	Working memory	MWM	↑ time spent in the previous quadrant and ↓ in the new one	Banqueri et al,^74^ 2018
MS	Daily,1 h from PND2 to PND9	Male Sprague–Dawley rats	Working memory	MWM	=	Huang et al,^75^ 2002
LBN	From PND2 to PND9	Male Sprague–Dawley rats	Working memory	MWM	↑ escape latencies	Ivy et al,^76^ 2010
LBN	From PND2 to PND9	Male Sprague–Dawley rats	Working memory	MWM	↓ time in the quadrant housing the platform on the previous day	Brunson et al,^71^ 2005
MS	Daily, 3 h from PND2 to PND21	Male Wistar rats	Working memory	MWM	↑ latency to find the hidden platform	Solas et al,^54^ 2010
LBN	From PND2 to PND9	Male and female C57B1/6J mice	Working memory	MWM	During the probe trial LBN males showed no preference over chance for the target quadrant (this effect did not appear in females)	Naninck et al,^73^ 2015
MS	Daily, 3 h from PND2 to PND14	Male Sprague–Dawley rats	Working memory	MWM	↓ time in the quadrant of the hidden platform	Dallé et al,^72^ 2017
LBN	From PND2 to PND9	Male 129S2/Sv X C57BL/6J mice	Working memory	MWM	=	Wang et al,^32^ 2011
LBN	From PND2 to PND21	Male Sprague–Dawley rats	Working memory	MWM	↓ time spent in target quadrant where the hidden platform was placed	Cui et al,^52^ 2006
MS	Daily, 3 h from PND1 to PND21	Male Sprague–Dawley rats	Working memory	MWM	↑ time in the target quadrant in which the platform was previously located	Zhang et al,^41^ 2014
MS	Daily, 3 h from PND1 to PND21	Male Wistar rats	Working memory	MWM	↑ distance looking for the platform on the first day of reversal learning phase	Xue et al,^78^ 2013
Domain: sensorimotor system						
MD	24 h from PND9 to PND10	Male and female Wistar rats	Innate motor patterns	OFT	↓ in walking and ↓ response to amphetamine	Ellenbroek et al,^86^ 2005
MS	Daily, 4.5 h from PND1 to PND21	Male and female Wistar rats	Innate motor patterns	OFT	↓ grooming behavior	Vivinetto et al,^39^ 2013
MS	Daily, 3 h from PND1 to PND10	Male and female Wistar rats	Innate motor patterns	OFT	MS female ↓ in the crossings number MS male ↑ in the crossings number	Borba et al,^55^ 2021
MD	Daily, 3 h from PND1 to PND10	Male and female Wistar rats	Innate motor patterns	OFT	MS male (but not female) ↓ rearing behavior	Abelaira et al,^56^ 2021
MD	Daily, 3 h from PND1 to PND10	Male Wistar rats	Innate motor patterns	OFT	↑ number of rearing at PND30	Réus et al,^57^ 2017
MD	Daily, 3 h from PND1 to PND10	Male Wistar rats	Innate motor patterns	OFT	=	Bertollo et al,^58^ 2024
MS	Daily, 3 h from PND1 to PND10	Male and female Wistar rats	Innate motor patterns	OFT	=	Andressa Caetano et al,^35^ 2024
MS	Daily, 6 h from PND2 to PND14	Male Wistar rats	Innate motor patterns	OFT	=	Montes-Rodríguez et al,^59^ 2024
MS	Daily, 3 h from PND2 to PND21	Male Sprague–Dawley rats	Innate motor patterns	OFT	↓ crossing and rearing	Liang et al,^44^ 2024
MS	Daily, 3 h from PND2 to PND21	Male and female Sprague–Dawley rats	Innate motor patterns	OFT	↓ rearing, wall-leaning and stretching, and ↑ grooming	Kim et al,^49^ 2020
ESS	3 mo	Male Long Evans rats	Motor actions (inhibition)	Go/no-Go	=	Hellemans et al,^68^ 2005
MS	Daily, 4 h from PND1 to PND10 (MS10) or from PND1 to PND21 (MS21)	Male Wistar rats	Sensorimotor dynamics	PPI	↓ startle amplitude during adolescence and adulthood	Banqueri et al,^37^ 2017
Domain: system for social processes						
MS	Daily, 3 h from PND1 to PND14	Male CD1 mice	Affiliation and attachment	SFC	Social investigation ↓ in control social fear conditioned mice during exposure to the first 5 social stimuli Social investigation ↓ in MS social fear conditioned mice only during the first 2 stimuli	Zoicas and Neumann,^34^ 2016
MS	Daily, 6 h from PND2 to PND14	Male Wistar rats	Affiliation and attachment	SIT	=	Montes-Rodríguez et al,^59^ 2024
MS	Daily, 6 h from PND4 to PND14	Male Wistar rats	Affiliation and attachment	SIT	= in sociability test ↑ time with the now familiar rat rather than with the novel rat in the social novelty test	Kambali et al,^46^ 2019
MS	Daily, 3 h from PND2 to PND14	Male Wistar rats	Affiliation and attachment	SIT	=	Rincel et al,^47^ 2019
MS	Daily, 3 h from PND1 to PND21	Male Sprague–Dawley rats	Affiliation and attachment	SDT	↑ time exploring the novel juvenile rat or the novel investigation index	Zhang et al,^41^ 2014
MS	Daily, 3 h from PND1 to PND14	Male CD1 mice	Affiliation and attachment	SDT	MS did not show the increased investigation of the novel vs the same rat during the social discrimination period	Zoicas and Neumann,^34^ 2016

5CSRTT, 5-choice serial reaction time task; ASST, attentional set-shifting test; CAT, cliff avoidance test; ELS, early life stress; EPM, elevated plus maze; ESI, early social isolation; FCT, fear conditioned test; FST, forced swimming test; FUST, female urine sniffing test; ESS, early social stress; LBN, limited bedding and nesting; MD, maternal deprivation; MS, maternal separation; MWM, Morris water maze; NOR, novel object recognition; OFT, open field test; OLT, object location task; PAT, passive avoidance task; PND, postnatal day; PPI, prepulse inhibition; PRSR, progressive ratio schedule of reinforcement; RAM, radial arm maze; rGT, rodent gambling task; SDT, social discrimination test; SFC, social fear conditioned; SIT, social interaction test; SPT, sucrose preference test; TST, tail suspension test; VCAT, virtual cliff avoidance test.

## 2 Effects of ELS on the negative valence system

One of the most studied consequences of ELS has been behaviors relating to negative emotional processes. The negative valence system has been defined as primarily responsible for responses to aversive situations or context, such as fear, anxiety, and loss.^24^^,^^87^^,^^88^ Here, 5 constructs can be defined as follows: acute threat; potential threat; sustained threat; loss; and frustrative nonreward. Animal research using the ELS procedure has mainly focused on acute threat, potential threat, and loss.

### 2.1 Acute threat

Responses to acute threat are related to the activation of the brain’s defensive motivational system to promote behaviors that protect the organism from perceived danger, and this construct is associated with fear.^24^^,^^87^^,^^88^ The paradigms used most frequently to assess acute threat are those related to fear conditioning, including the fear conditioned test (FCT), passive avoidance test, step-down inhibitory avoidance task, and the shuttle box escape test. In relation to the FCT, animals learn to associate either a discrete stimulus (eg, an auditory conditioned stimulus) or surrounding context with an aversive unconditioned stimulus, such as mild electric shocks, which, following conditioning, elicits freezing behavior.^89^^,^^90^ Despite considerable research, however, no clear consensus has been reached on the effects of ELS on fear conditioning, with studies showing enhanced conditioned fear,^27^^,^^29^^,^^33^ attenuated conditioning,^28^^,^^30^, ^31^, ^32^^,^^36^ or no effect.^26^^,^^33^, ^34^, ^35^^,^^38^ Similar results were found using avoidance paradigms, where a conditioned or unconditioned response is present when animals must inhibit a motor response toward a dangerous zone.^91^ The key variable measured is avoidance latency, which refers to the time taken for the animal to move into the dangerous zone. However, in general, MS stress has no obvious effect on passive and active avoidance learning to aversive stimuli.^37^^,^^39^ Thus, acute threat responses are not robustly affected by ELS.

### 2.2 Potential threat

Responses to potential threat refers to the activation of a brain system in which harm may potentially occur but is distant, ambiguous, or low/uncertain in probability, characterized by a pattern of responses such as enhanced risk assessment.^24^^,^^87^^,^^88^ Relevant tasks to assess potential threat responses in rodents include the elevated plus maze (EPM), light/dark test, and open field test (OFT). The EPM consists of 4 elevated arms that radiate from a central platform, forming a plus shape. Two of the opposed arms are walled and therefore safe, whereas the remaining 2 arms are open and potentially threatening.^92^ In the actual test, a rodent is placed in the central area, and then, it is left to explore the maze for a defined but relatively short period. The amount of time spent in the walled arms is compared with the amount of time spent in the open arms as a measure of anxiety or fear.^93^ However, similar to the mixed findings reported earlier for actual threats, ELS does not appear to uniformly affect responses to potential threats with reports of increased anxiety,^40^^,^^42^^,^^44^ reduced anxiety,^41^^,^^43^^,^^45^ or no effect of MS on the EPM.^33^, ^34^, ^35^^,^^38^ However, a recent meta-analysis concluded that EPM is not a sensitive test of anxiety-like behavior in adult rats with a history of ELS.^94^ Of note, the EPM is subject to considerable procedural variability in how the test is implemented from one laboratory to the next with differences in strain, age, and time of testing relative to the diurnal cycle.^95^ Nevertheless, in relation to the light/dark test, MS animals show higher anxiety levels measured by an increased time in the dark chamber together with a lower latency to enter into the dark chamber^47^ and decreased time spent in the light chamber.^47^ In the OFT, the typical test area is circular or square with an area large enough, based on the size of the subject tested, to elicit a feeling of openness in the center of the maze. Ambulation and time spent in the center of the arena are routinely assessed as indices of anxiety.^96^ A small number of studies that implemented the OFT support the link between MS and increased anxiety.^42^^,^^44^^,^^50^ However, other studies report anxiolytic effects of MS stress,^43^^,^^45^^,^^49^ while the LBN procedure in mice reportedly had no effect on the relative time spent in the central area of the chamber.^48^

### 2.3 Loss

The psychological construct of loss within the negative valence system describes a state of deprivation of a motivationally significant con-specific, object, or situation and can lead to the emergence of so-called learned helplessness behaviors.^24^^,^^87^^,^^88^ The procedures used to assess loss include the forced swimming test (FST) and tail suspension test, with which to measure learned helplessness by immobility and climbing behavior^97^^,^^98^; the sucrose preference test, to measure voluntary access to highly palatable food test; and an ecological test to measure anhedonia called the female urine sniffing test to measure anhedonia.^62^^,^^99^^,^^100^ Research has generally shown that ELS in the form of MS can increase the expression of helplessness-like behaviors in the FST and tail suspension test,^38^^,^^40^^,^^49^^,^^51^^,^^54^^,^^55^^,^^57^^,^^58^^,^^60^ which also extends to the LBN procedure.^52^ A minority of studies, however, report no effect of ELS on the FST in either rats^53^^,^^59^ or mice.^32^ With respect to anhedonia, the most used tests measure the animals’ preference for stimuli with different hedonic values. MS^47^^,^^60^ and LBN^62^ animals show a reduced preference for hedonic stimuli, including sucrose on the sucrose preference test,^47^^,^^60^ palatable food,^62^ and female odors.^47^

Thus, overall, MS has mixed effects on behaviors relevant to the negative valence system with no clear effects on anxiogenic behaviors elicited by acute or potential threats. MS animals appear, however, to be more susceptible to helplessness and anhedonia, consistent with depression-like behavior.

## 3 Effects of ELS on the positive valence system

In contrast to the negative valence system, less research has been carried out on the effects of ELS on the positive valence system domain. This domain is primarily responsible for responses to positive motivational stimuli and contexts and includes constructs that promote seeking rewarding experiences, the positive emotions associated with them, and the use of reward to facilitate learning and habit formation.^24^^,^^87^^,^^88^

### 3.1 Reward learning

Reward learning defines a process whereby organisms acquire information about stimuli, actions, and contexts that predict positive outcomes. It also includes behavior that is modified when a novel reward occurs or when outcomes are better than expected, and it is a type of reinforcement learning.^24^^,^^87^^,^^88^ One task used to assess reward learning is the rodent gambling task (rGT), an analog of the Iowa gambling task. This task allows animals to choose among 4 options, signaled by the illumination of 4 response apertures, each with a unique probability of sucrose reward or time-out punishment.^101^ As in the Iowa gambling task, the best strategy is to favor options associated with not only smaller incremental gain but also smaller penalties, resulting in a maximum number of sugar pellets.^102^ In a notable study, MS rats performed suboptimally on the rGT and were overly represented in the poor decision-making category compared with control rats.^42^ The attentional set-shifting task is a learning paradigm used to assess reward-seeking behavior that is motivated by perceptual discriminative stimuli.^103^ Subjects are trained to associate a specific digging medium in a ceramic bowl with a particular food reward such as chocolate-flavored cereal hidden at the bottom of the bowl. Correct and incorrect responses to different digging media are recorded and used to assess attentional discriminative performance to single or compound discriminative stimuli with reversals in stimulus-reward contingencies presented to tax attentional flexibility. Although animals deprived of material care in early life were not impaired on simple discriminative performance, impairments emerged for more complex discriminative trials involving compound discrimination, stimulus-reward reversals, and extra-dimensional set shifting.^63^

Reward learning can also be inferred by self-administration procedures.^65^ Animals subjected to early MS^66^ and LBN stress^62^ show higher rates of methamphetamine self-administration^66^ and faster acquisition of cocaine self-administration compared with control animals.^98^ MS animals also show higher rates of alcohol consumption.^60^ However, LBN rats self-administer a lower number of heroin infusions during the loading phase and exhibit a reduced hedonic setpoint for opioids.^104^

### 3.2 Reward valuation

Within the positive valence system domain, the construct of reward valuation relates to reward delay and effort and is influenced by pre-existing biases, learning, memory, stimulus characteristics, and deprivation states.^24^^,^^87^^,^^88^ A recent study found that ELS in the form of wet bedding and nesting led to increased latencies to collect reward in the presence of fear-associated cues together with greater suppression during a conditioned suppression task.^67^ The most widely used tasks to measure reinforcement devaluation are those where animals must choose between a small immediate reinforcement and a large, delayed reinforcement.^105^ Rats isolated from social interaction at young age show reduced impulsivity on a delayed reinforcement task (ie, show a higher preference for delayed larger magnitude rewards)^68^ and temporal reward discounting task.^69^ However, early social isolation does not apparently affect the motivation to self-administer alcohol on a progressive ratio schedule of reinforcement.^64^

Taken together, these findings suggest that MS stress causes a blunting of behavior within the positive valence system. By maintaining higher rates of drug self-administration, a preference for higher magnitude rewards, and slower food collection latencies, MS stress may impair the reward valuation system, thereby affecting self-regulatory behaviors motivated by food and drug incentives.

## 4 Effects of ELS on the cognitive system

The cognitive system domain within the RDoC framework is responsible for processing information and comprises various cognitive processes such as attention, perception, memory, language, cognitive control, and working memory.^24^^,^^87^^,^^88^ However, most studies using ELS procedures in rodents have focused on the long-term consequences of ELS on cognitive control and memory.

### 4.1 Cognitive control

Cognitive control modulates other cognitive and emotional processes to support goal-directed behavior, especially when dominant or automatic responses are insufficient to meet the demands of the current context. It is also recruited in novel situations, where appropriate actions must be selected from among competing alternatives.^24^^,^^87^^,^^88^ Tasks used to investigate cognitive control in rodents include the 5-choice serial reaction time task (5CSRTT), the cliff avoidance test, and the visual cliff avoidance test. The 5CSRTT assesses sustained and divided attention as well as inhibitory response control.^106^ MS^107^ and isolation reared^69^ animals showed increased perseverative responses on the 5CSRTT during the baseline and the stimulus duration (0.5-second) challenge^107^ and during task acquisition.^69^ MS rats also exhibited fewer omissions compared with the control group.^107^ Although avoidance tasks have been used to measure emotional reactions, they may also reflect a lack of inhibitory control when rodents move quickly in a threatening environment. In a recent study, it was reported that MS rats show behavior consistent with increased impulsivity on cliff avoidance test and visual cliff avoidance test,^43^ by measuring an animal’s tendency to engage in risk-taking behavior by failing to adequately inhibit actions that lead to falls from an elevated platform. Taken together, these findings suggest that ELS affects response inhibitory control resulting in increased perseveration and impulsive behavior in certain contexts.

### 4.2 Spatial memory

Declarative memory is the acquisition or encoding, storage and consolidation, and retrieval of representations of facts and events. Declarative memory provides the critical substrate for relational representations (ie, for spatial, temporal, and other contextual relations among items).^24^^,^^87^^,^^88^ Studies on ELS have assessed spatial memory on the Morris water maze (MWM),^108^ the radial arm maze,^109^ and the object location task (OLT).^110^ In the MWM, rodents are released from different starting points and learn to use distal cues to navigate a direct path to a spatially fixed hidden platform.^111^ The effects of ELS on spatial memory are mixed. For example, a range of studies report deficits on the MWM (increased latencies) in rats^52^^,^^71^^,^^76^ and mice^32^^,^^48^^,^^73^ exposed to LBN. Deficits in spatial memory have also been reported in MS rats.^72^^,^^75^^,^^77^ However, other studies report no effect of MS on MWM performance.^31^^,^^41^^,^^54^^,^^74^^,^^78^ On the radial arm maze, MS animals showed an increased percentage of correct responses during spatial learning.^46^ In the OLT, animals initially explore a maze in the absence of an object. Subsequently, during the training period, animals are placed in the arena with 2 identical and familiar objects. Animals are then given a retention test, where one object is placed at the same location as the training session, whereas the other similar object is placed in a novel location.^111^ Mice exposed to ELS learned the location discrimination but overall showed a decreased localization index in LBN mice^73^^,^^79^, ^80^, ^81^, ^82^ and MD rats.^81^

### 4.2 Nonspatial memory

A widely used procedure to assess nonspatial memory is the novel object recognition (NOR) test. In this test, the rodent is presented with 2 similar objects during the first session. One of the 2 objects is then replaced by a new object during a second session. The amount of time taken to explore the new object provides an index of recognition memory.^112^^,^^113^ Many studies report deficits in this form of learning following ELS. For example, NOR is impaired in MS rats,^37^^,^^51^^,^^54^ LBN rats,^76^^,^^83^ MS mice,^32^^,^^84^^,^^85^ and LBN mice.^48^^,^^73^ However, discrepant null findings have been reported in rats^39^^,^^53^^,^^81^ and mice^34^ exposed to MS.

### 4.3 Working memory

Working memory is the active maintenance and flexible updating of goal/task relevant information that has limited capacity and resists interference. These representations may involve flexible binding of representations, be characterized by the absence of external support for the internally maintained representations, and be relatively temporary. It involves active maintenance, flexible updating, limited capacity, and interference control.^24^^,^^87^^,^^88^ To assess updating of working memory, the last stage of the MWM is often used whereupon the platform is moved, and swimming latencies are recorded.^111^ Animals exposed to ELS show impairments in updating the new platform location and perseverate in the previous quadrant compared with the control group.^41^^,^^74^ Rats exposed to LBN also exhibited longer escape latencies during the probe session compared with control group,^76^ a deficit also observed in MS rats.^54^^,^^78^ However, a small of numbers report null effects of LBN^32^ and MS^75^ on this task.

Overall, within the cognitive system, MS rats show deficient inhibitory control as reflected by increased perseveration and impulsivity in specific contexts. However, effects on hippocampal- and non–hippocampal-dependent learning and memory processes are less robust except for impaired NOR, which may involve deficits in the updating of working memory.

## 5 Effects of ELS on the sensorimotor system

The sensorimotor system underpins the control and execution of motor behaviors and their refinement during learning and development. There are 4 different RDoC constructs in this domain: (1) motor actions (with 5 subconstructs; action planning and selection, sensorimotor dynamics, initiation, execution, and inhibition and termination); (2) agency and ownership; (3) habit-sensorimotor, and (4) innate motor patterns. However, most ELS studies have only investigated innate motor patterns and sensorimotor dynamics.

### 5.1 Motor actions

Motor actions are processes that regulate the suppression of motor plans, either prior to or following the initiation of an action, and include selection, sensorimotor dynamics, initiation, execution, and termination.^24^^,^^87^^,^^88^ In general, motor inhibition can be effectively assessed using go/no-go discrimination tasks, where rats must learn to perform an action in response to a specific cue (go signal) and to withhold that action when a different cue (no-go signal) is presented.^114^ Rats subjected to ELS in the form of early isolation rearing were not impaired on this type of task compared with control animals.^68^

### 5.2 Innate motor patterns

Innate motor patterns are unlearned action plans that may be triggered by internal or external stimuli. This can include such behaviors as stereotyped expressions of affect, orientation to salience, innate approach and withdrawal phenomena, and startle responses.^24^^,^^87^^,^^88^ In preclinical research, motor behaviors are often assessed using the OFT. This test, described in the negative valence system earlier, provides a measure of potential threat by the time spent not only in the center of the arena but also other types of motor behaviors. Numerous studies highlight alterations in innate motor patterns on this test after ELS in rats. Thus, MS predates a reduction in ambulatory behavior in adult rats,^49^^,^^86^ both decreased^39^ and increased^49^ grooming behavior, increased rearing behavior,^57^ and a reduced response to the psychomotor stimulation effects of amphetamine.^86^ However, null findings have also been reported on the OFT.^35^^,^^58^^,^^59^

### 5.3 Sensorimotor dynamics

Sensorimotor dynamics are processes involved in the specification or parameterization of an action plan and program based on the integration of internal or external information, such as sensations and urges and modeling of body dynamics.^24^^,^^87^^,^^88^ One of the most widely used paradigms to assess sensorimotor dynamics is prepulse inhibition (PPI). PPI is a translational paradigm for the assessment of attention-processing deficits in patients with schizophrenia and other psychotic disorders. PPI of the acoustic startle reflex refers to the reduction in the startle magnitude to a startling acoustic pulse stimulus when it is shortly preceded by a weaker prepulse stimulus, which is typically below the threshold for generating its own startle reaction.^115^^,^^116^ In a recent study, a short 10-day period of MS reduced startle amplitude but not when the separation period was extended to 21 days.^37^ The authors interpreted these findings as suggestive of differential effects of the short separation period on the brain dopaminergic systems with heightened activity enabling better predictions of the startling pulse.

In summary, the nascent evidence available for the sensorimotor system suggests that MS animals are not impaired in motor inhibition tasks but do show reduced spontaneous and stimulant-elicited behaviors in an open field environment. Thus, effects of MS stress may be limited to more cognitive (decisional) forms of impulsive behavior rather than motor control per se. More broadly, ELS may affect motivational arousal processes that indirectly affect the sensorimotor system, consistent with more direct impacts on the positive valence system, as discussed previously.

## 6 Effects of ELS on social processing

Systems for social processes mediate responses in relationship settings of various types, including perception and interpretation of the actions of other individuals.^24^^,^^87^^,^^88^

### 6.1 Affiliation

Affiliation is engagement in positive social interactions with other individuals and is moderated by the processing of social cues and social motivation, manifesting itself most obviously in social approach behaviors. Affiliation is a behavioral consequence of social motivation and can manifest itself in social approach behaviors.^24^^,^^87^^,^^88^ Different social paradigms have been used to measure social learning and discrimination related to ELS exposure. Using the social FCT, Zoicas and Neumann^34^ found that mice exposed to MS were faster to extinguish social fear without impairing its acquisition or expression. Thus, MS may help to strengthen coping responses to stressful social encounters later in life. Other research has found that MS rats spend more time exploring unfamiliar juvenile rats compared with control rats.^41^ However, other studies report no effect of MS on the sociability measure of the social interaction test^46^^,^^47^^,^^59^ or greater investigation of familiar rather than novel rats on the social novelty test of the social interaction test.^46^ Given the paucity of studies, no firm conclusions can yet be drawn on the effects of ELS on social processing.

## 7 ELS and psychopharmacological interventions

In this section, we discuss how ELS affects the psychopharmacological effects of a range of compounds used to treat stress-related disorders such as depression and anxiety. Given the available empirical evidence, we have restricted our analysis to interventions targeting the glutamatergic, serotonergic, and endocannabinoid (ECB) systems, as summarized in Table 2.^117^, ^118^, ^119^, ^120^, ^121^, ^122^, ^123^, ^124^, ^125^, ^126^, ^127^, ^128^, ^129^, ^130^, ^131^, ^132^, ^133^ A recurring theme of this analysis is that ELS causes robust and persistent changes in behavior and neurobiological processes, which are often differentially affected by pharmacological agents in control and ELS animals. The studies discussed in further sections illustrate this point. However, in some of these studies, nonstressed control animals only received a vehicle injection rather than the active pharmacological agent.^56^^,^^117^^,^^119^^,^^123^ Thus, it remains an open question whether the reported effects in these studies were dependent on ELS. In the remaining studies, this was the case, with differential effects of pharmacological interventions observed between control and ELS animals.

**Table 2:** Neuropsychopharmacological modulation of the behavioral and neurobiological consequences of early life stress

ELS Model	ELS Protocol	Drug	Behavioral Effects	Neurobiological Effects	Citation
Glutamatergic interventions					
MD	Daily, 3 h from PND1 to PND10	Ketamine (15 mg/kg, i.p.)	Males: ↓ immobility, ↑ swimming FST; ↓ rearings OFT; females: = FST= OFT.	Males and females: reversed ↑ lipid peroxidation (TBARS) in the PFC and hippocampus; ↓ protein carbonylation in the hippocampus; reversed ↑ in nitrite/nitrate concentration in the PFC; ↓ SOD and CAT activity in the PFC and hippocampus; reversed ↑ in MPO activity in the hippocampus. Only in males: reversed ↑ in IL-6 levels in the hippocampus.	Abelaira et al,^56^ 2021
MS	Daily, 3 h from PND1 to PND10	Ketamine (15 mg/kg, i.p.) twice a week	Males: ↓ immobility time in FST at PND61	BBB integrity: ↓permeability in females (PFC at PND21) and males (PFC at PND21, PND41, PND61; hippocampus at PND41). Reversed BBB damage in females (PFC and hippocampus at PND61). Oxidative stress: ↓ TBARS levels in females (PFC and serum at PND21) and males (PFC at PND21, PFC and serum at PND61)	Carlessi et al,^117^ 2022
MD	Daily, 3 h from PND1 to PND10	Single dose of *S*-ketamine (15 mg/kg) at PND 46	↓ immobility time in FST and ↑ grooming time in the Splash test. = locomotor activity, = crossing or rearing in the OFT.	↓ protein damage in the hippocampus, amygdala and NAcc: ↓ carbonyl protein levels. ↓ lipid damage in the hippocampus, amygdala, and nucleus accumbens: ↓ MDA levels. ↑ antioxidant enzyme activity in the hippocampus, amygdala, and nucleus accumbens: ↑ SOD activity. ↑ antioxidant enzyme activity in the hippocampus and nucleus accumbens: ↑ CAT activity.	Réus et al,^118^ 2015
MD	Daily, 3 h from PND1 to PND10	Ketamine (5 mg/kg, i.p., twice/week), escitalopram (5 mg/kg, oral, daily), ECS (3 times/week)	Males: (escitalopram, ECS, ketamine, ECS + escitalopram, ECS + ketamine, and ECS + escitalopram + ketamine) ↓ immobility FST. (All treatments, except ECS + escitalopram + ketamine) ↑ grooming time splash test. (ECS, ECS + escitalopram, and ECS + escitalopram + ketamine) ↓ rearings in the OFT. Females (ECS + escitalopram, ketamine + escitalopram, and ECS + escitalopram + ketamine) ↓ immobility FST. (Escitalopram, ketamine, ECS + escitalopram, ketamine + escitalopram, and ECS + escitalopram + ketamine) ↑ grooming splash test.	Escitalopram: Males ↓ lipid peroxidation (TBARS), protein carbonylation, ↓ MPO activity in the PFC, hippocampus, and serum; ↓ IL-1*β* and TNF-*α* levels in the PFC of both sexes, ↓ IL-6 levels in the PFC and hippocampus of females. ECS: ↑ CAT activity in the PFC of females, ↓ lipid peroxidation and nitrite/nitrate concentration in the PFC of males, ↓ MPO activity in the PFC and hippocampus of both sexes, ↓ IL-1*β* levels in the PFC of both sexes, ↓ IL-6 levels in the PFC of males and the hippocampus of females, ↓ TNF-*α* levels in the PFC of males. Ketamine:↑ CAT in the PFC of males when combined with ESC, ↓ lipid peroxidation in the PFC and hippocampus of males, ↓ MPO activity in the PFC of females and the PFC and serum of males, ↓ IL-1*β* levels in the PFC of both sexes, ↓ IL-6 levels in the PFC and hippocampus of females, ↓ TNF-*α* levels in the PFC of females.	Abelaira et al,^119^ 2022
Social stress	Pup placed with a resident adult CD1 male mouse. Daily, 30 min from PND14 to PND21	Minocycline (50 mg/kg)		Males: ELS ↓ DAT and TH expression in the VTA. Minocycline prevented ELS-induced changes in DA neuron morphology and DAT and TH expression.	Catale et al,^120^ 2022
Serotoninergic interventions					
MS	Daily, 3 h from PND1 to PND10	Escitalopram (10 mg/kg)	Males: ↑ immobility time in FST at PND61. Males: ↓ swimming time in the FST Females: No significant changes in immobility time in the FST.	BBB integrity: ↓ permeability in females (PFC and hippocampus at PND21) and males (PFC at PND21, PND41). Reversed BBB damage in females (PFC and hippocampus at PND61). Oxidative stress: ↓TBARS levels in males (PFC and hippocampus at PND21) and females (PFC at PND61). ↑ SOD activity in females (hippocampus at PND41). ↑ CAT activity in females (PFC at PND41).	Carlessi et al,^117^ 2022
MS	Daily, 3 h from PND2 to PND14	Escitalopram (25 mg/kg per day)	↑ latency in the FST. ↑ time spent on the platform quadrant MWM probe trial.		do Couto et al,^121^ 2012
Prenatal stress	Pregnant females were subjected to 3 stress sessions (45 min) daily from 14th day of pregnancy to delivery	Fluoxetine (10 mg/kg per day)	↓ immobility and ↑ swimming time in the FST.	↑ Cathepsin D: role in neuronal cell homeostasis and the degradation of unfolded or oxidized proteins. ↑ Stathmin-1 (STMN1): involved in learning processes, neurite outgrowth, and synaptic plasticity. ↑ Dynamin-1 (Dnm-1): involved in endocytosis and neurotransmission. ↑ Protein DJ-1: involved in protecting against oxidative stress and promoting mitochondrial biogenesis.	Glombik et al, 2017^122^
Prenatal stress	Pregnant females were subjected to 3 stress sessions (45 min) daily from 14th day of pregnancy to delivery.	Imipramine (10 mg/kg per day)	↓ immobility and↑ swimming time in the FST.	↓ 14-3-3 protein epsilon: This protein is involved in synaptic function, learning and memory. Its downregulation may be related to imipramine’s therapeutic effects. ↓ Cytochrome b-c1 complex subunit 2 (complex III): involved in the mitochondrial respiratory chain, ↑ COP9 signalosome complex subunit 4: involved in cell cycle regulation, signal transduction, and autophagy.	Glombik et al, 2017^122^
LBN	From PND7 to PND14	Paroxetine (5 mg/kg, i.p.; PND 45-60)	No prevention of ELS-induced depressive-like phenotype.	↓ miR-135^a^ Expression in the mPFC, CA1, LHb, and DR in males. ↑ miR-16 Expression in the LHb of males and the DR in both males and females. ↓ faah Expression in the mPFC of males.	Portougalov et al, 2022^128^
MS	Daily, 3 h from PND2 to PND14	*Bifidobacterium infantis*; Citalopram	↓ immobility time in FST	↓ IL-6 response; Restored NA levels	Desbonnet et al,^123^ 2010
MS	Daily, 3 h from PND2 to PND14	Ketanserin (5 mg/kg)	↓ anxiety in OFT and EPM tests	Normalized Arc mRNA regulation in the PFC. Prevents ↑ of Prkcb1, Plek, and Ppp3ca mRNA and ↑ 5-HT2A mRNA levels in the PFC.	Benekareddy et al,^124^ 2011
ESS	Single housed from PND21 to PND23	Buspirone (0.5 mg/kg); Desimipramine (2.5 mg/kg) 2-wk daily	Buspirone but not desimipramine induced time-dependent increase in premature responses on 5-CSRT task not observed in ESS rats		Tung et al,^70^ 2023
ESS	Single housed from PND21 to PND23	D-amphetamine; acute (0.4, 0.6, and 0.8 mg/kg)	Less impulsive action and choice on the 5-CSRT task and a temporal discounting reward task		Liu et al,^69^ 2017
MS	Daily, 3 h from PND5 to PND21	GPR55 agonist O-1602 (0.4 mg/kg and 2.0 mg/kg) i.p. for 5 d at PND42	Enhanced performance in Novel NORT, Y-maze, and MWM tests.	↑ GPR55 and TPH2 expression in the DRN: enhanced 5-HT synthesis and release. ↑ frequency and amplitude of miniature excitatory postsynaptic currents (mEPSCs) in DRN 5-HT neurons. Upregulated NMDAR (GluN2A and GluN2B) and AMPAR (phosphorylated GluA1) expression in the DRN.	Sun et al,^125^ 2024
MS	Daily, 3 h from PND5 to PND21	TPH2 knockdown using adenoassociated virus injection into the DRN	↓ performance in NORT and MWM tests. ↓ beneficial effects of O-1602 treatment: TPH2 knockdown diminished the improvements in learning and memory seen by O-1602	↓ TPH2 expression in the DRN: ↓ 5-HT synthesis	Sun et al,^125^ 2024
Cannabinoid interventions					
LBN	From PND7 to PND14	WIN55, 212-2 (1.2 mg/kg, i.p.)	In both sexes: late-adolescence (P45-P60): ↑ spatial, social and object recognition memory, ↓anxiety In males: adolescence (P30-P45): = spatial and social recognition memory and ↓anxiety.	Late adolescence (P45-P60): in males, ↑ BLA-CB1r and ↓ infralimbic -PFC-GRs. In females, normalized CA1-CB1r and infralimbic-PFC-GRs.	Alteba et al,^126^ 2016
LBN	Limited “Sunny chips” bedding material from PND7 to PND14	URB597 (0.4 mg/kg, i.p.), JZL184 (2 mg/kg, i.p)	URB597 and JZL184 prevented in both sexes: ↓ social interaction at P23. In adulthood, ↓ social preference and social recognition. ↑ immobility in the FST.	URB597 and JZL184 prevented ↓ MAGL activity and BDNF, impairment of LTP and baseline synaptic activity in both sexes.	Alteba et al,^127^ 2020
LBN	From PND7 to PND14	URB597 (0.4 mg/kg, i.p.) at PND45–60	Improved ↑ motor activity in males. ↑ social preference in females. ↑ social recognition and ↓ immobility in the FST in both sexes.	↑ miR-135^a^ Expression in the mPFC of females. ↑ miR-16 Expression in the mPFC of males. ↓ cnr2 Expression in the mPFC of females. ↑ faah Expression in the mPFC of females.	Portougalov^128^ et al, 2022
Other interventions					
MS	Daily, 3 h from PND2 to PND14	MLCK inhibitor; ML-7, 5 mg/kg daily i.p. PND2-14	Prevented MS-induced sexual reward-seeking impairment	Reversed corticosterone response to stress and normalized the abundance of members in the gut such as Lachnospiraceae, Clostridiales, *Desulfovibrio*, Bacteroidales, Enterorhabdus, and *Bifidobacterium.*	Rincel et al,^47^ 2019
MS	Daily, 3 h from PND1 to PND10	Probiotic (*Bifidobacterium infantis*): administered orally at 1 × 10^10^ CFU diluted in 100 mL of water once a day.	↓ immobility in the FST in females, not in males.	↓ BBB permeability in females (hippocampus at PND21) and males (PFC at PND21, PND61). Reversed BBB damage in females (PFC at PND41, PFC and hippocampus at PND61). Oxidative stress: ↓ TBARS levels in both sexes (PFC and serum at PND21, PFC, hippocampus, and serum at PND41 and PND61). ↑ SOD activity in females (PFC, hippocampus, and serum at PND41) and males (PFC at PND41).	Carlessi et al,^117^ 2022
MS	Daily, 3 h from PND1 to PND21	Intranasal OT (2 *μ*g/*μ*L, every other day for 7 d from PND22 to PND34)	↑social interaction. ↑ social novelty. ↓ anxiety-like behavior, ↓ grooming behavior in the OFT. ↑ total distance ↑ velocity in the OFT. ↑ spatial learning by ↓ escape latency and path length in the MWM. ↑memory retention, more time and distance spent in the target quadrant during MWM probe trial. = discrimination ratio in the NOR test.	Restored the ability of LTP induction in the CA1 area of the hippocampus. OT did not reduce the elevated corticosterone levels induced by MS.	Joushi et al,^129^ 2021
MS	Daily, 3 h from PND1 to PND21	Intranasal OT (2 *μ*g/*μ*L, every other day for 7 d from PND22 to PND34)	↑ prosocial behavior in the double T-maze prosocial choice task. ↑ percentage of choices to rewards for both them and a partner rat. ↑ social bias score: greater difference in choices favoring mutual rewards in the presence of a partner rat compared with a toy rat.		Joushi et al,^130^ 2022
MS	Daily, 3 h from PND1 to PND21	EE (PND22–34) + intranasal OT (2 *μ*g/*μ*L, every other day for 7 d from PND22 to PND34)	↑ prosocial behavior: enhanced mutual reward preferences in the double T-maze task. ↑ percentage of choices rewards for themselves and their partners.		Joushi et al,^131^ 2022
MS	Daily, 3 h from PND1 to PND21	Intranasal OT (2 *μ*g/*μ*L, every other day for 7 d from PND22 to PND34)	Improved decision making: ↑ percentage of advantageous choices in the rGT		Joushi et al,^131^ 2024
MS	Daily, 3 h from PND1 to PND21	EE from PND22 to PND34	Improved decision making: ↑ percentage of advantageous choices in the rGT		Joushi et al,^131^ 2024
MS	Daily, 3 h from PND1 to PND21	Combined EE and OT	No improvements in decision making compared with either treatment alone in the rGT		Joushi et al,^131^ 2024
MD	Daily, 3 h from PND1 to PND10	EE for 10 d starting at PND21 (tested at PND31)	↑ open arm time in EPM in males. ↓ memory habituation in females in the OFT	↓ lipid damage: ↓ TBARS levels in males. ↑ antioxidant capacity in females: ↑ sulfhydryl content. ↓ antioxidant enzyme activity in both sexes: ↓ CAT	Réus et al,^132^ 2023
MD	Daily, 3 h from PND1 to PND10	EE for 20 d starting at PND 21 (evaluated at PND 41)	↑ open arm time and entries in EPM in females. Restored memory habituation in females in the OFT	↓ oxidative stress in males: ↓ carbonyl protein ↓ lipid damage in males: ↓ TBARS levels. ↑ antioxidant capacity in both sexes: ↑ sulfhydryl concentration. ↑ antioxidant enzyme activity in females: ↑ SOD. ↓ antioxidant enzyme activity in males: ↓ SOD activity. ↑ antioxidant enzyme activity in both sexes: ↑ CAT	Réus et al,^132^ 2023
MD	Daily, 3h from PND1 to PND21	EE for 40 days starting at PND 21 (evaluated at PND 61)	↑ open arm time and entries in EPM in females. Restored memory habituation in both sexes in the OFT	↓ protein damage in males: ↓ carbonyl protein levels. ↓ lipid damage in males: ↓ TBARS levels. ↑ antioxidant capacity in both sexes: ↑ sulfhydryl concentration. ↓ antioxidant enzyme activity in females: ↓ SOD and in males: ↑ CAT	Réus et al,^132^ 2023
Single-prolonged stress	Restraint, forced swimming, and anesthesia at PND30.	Paeoniflorin (PF; 40 mg/kg) administered orally once daily for 4 wk, from PND38 to PND65	↑ total distance in the OFT. ↑ percentage open arm entries and time in the EPM ↓ escape latency in the MWM test, ↑ target quadrant time and distance, ↑ platform crossings in the MWM probe test. ↓ freezing behavior in both the contextual and cued fear conditioning tests.	Modulation of HAT/HDAC ratio in the hippocampus and amygdala: ↓ HAT activity and ↑ HDAC activity in the hippocampus, leading to a lower HAT/HDAC ratio. ↑ HAT activity and ↓ HDACs in the amygdala, ↑ HAT/HDAC ratio.	Xu et al,^133^ 2024

5-HT, serotonin; 5-HT_2A_, serotonin type 2A receptor; AMPAR, *α*-amino-3-hydroxy-5-methyl-4-isoxazolepropionic acid receptor; Arc, activity-regulated cytoskeletal-associated protein; BBB, blood–brain barrier; BDNF, brain-derived neurotrophic factor; BLA, basolateral amygdala; CAT, catalase; CB1, cannabinoid receptor subtype 1; cnr2, endocannabinoid target; ConA, concanavalin A, a T cell stimulant; DA, dopamine; DAT, dopamine transporter; Dnm1, dynamin-1; DR, dorsal raphe; DRN, dorsal raphe nucleus; ECS, electroconvulsive stimulation; EE, environmental enrichment; ELS, early life stress; EPM, elevated plus maze; faah, endocannabinoid target; ESS, early social stress; FST, forced swimming test; GPR55, G-protein-coupled receptor 55; GR, glucocorticoid receptor; H3K9, lysine 9 residue of histone H3; H4K12, lysine 12 residue of histone H4; HAT, histone acetyltransferase; HDAC, histone deacetylase; HPA, hypothalamus-pituitary-adrenal; i.p. intraperitoneal; IL, interleukin; IR, isolation rearing; LBN, limited bedding and nesting; LHb, lateral habenula; LTP, long-term potentiation; MAGL, monoacylglycerol lipase; MD, maternal deprivation; MDA, malondialdehyde; mEPSC, miniature excitatory postsynaptic current; miR, microRNA; MPO, myeloperoxidase; MS, maternal separation; MWM, Morris water maze; NA, noradrenaline; NAcc, nucleus accumbens; NMDAR, *N*-methyl-d-aspartate receptor; NOR, novel object recognition; OFT, open field test; OT, oxytocin; PF, paeoniflorin; PFC, prefrontal cortex; Plek, pleckstrin; PND, postnatal day; Ppp3ca, protein phosphatase 3, catalytic subunit, *α* isoform (calcineurin A *α*); Prkcb1, protein kinase C *β*1; PTSD, posttraumatic stress disorder; rGT, rodent gambling task; SN, substantia nigra; SOD, superoxide dismutase; SPS, single-prolonged stress; SPT, social preference test; STMN1, stathmin-1; TBARS, thiobarbituric acid reactive substance; TH, tyrosine hydroxylase; TNF, tumor necrosis factor; TPH, tryptophan hydroxylase; VTA, ventral tegmental area.

## 8 Glutamatergic interventions

A myriad of studies have investigated the effects of the NMDA receptor antagonist, ketamine in rodents exposed to ELS. Ketamine and its derivatives are widely recognized for their rapid and robust antidepressant effects.^134^ The studies reviewed mainly focus on the effects of ELS on the loss construct of the RDoC negative valence system domain. In several recent studies ketamine has been shown to reverse depressive-like behaviors in male rats subjected to MD and MS forms of ELS. For example, ketamine reduced immobility time in the FST,^56^^,^^117^, ^118^, ^119^ a widely used measure of behavioral despair. These findings suggest that ketamine increases motivation and active coping strategies in the face of stress. However, in female rats exposed to MD, ketamine alone had no effect on immobility time in the FST. Only when combined with the selective serotonin reuptake inhibitor (SSRI) escitalopram or electroconvulsive stimulation (ECS) were significant effects of ketamine observed in the FST, indicating an enhanced antidepressant action.^119^ Moreover, ketamine increased grooming time in the splash test in both sexes in rats exposed to MD, indicating a reversal of anhedonia, a core symptom of depression characterized by a loss of self-care and motivation.^118^^,^^119^ This rescue effect of ketamine on anhedonia was also observed when combining ketamine with ECS or when combining ketamine with escitalopram. Notably, the administration of ketamine did not significantly alter motor behaviors, measured by spontaneous locomotor activity of male and female rats exposed to MD in the OFT, suggesting that its effects on behavior are specific to depressive-like symptoms rather than motor function.^118^^,^^119^

Beyond primary effects mediated by the NMDA receptor, recent evidence indicates the beneficial behavioral effects of ketamine may be related to the reduction in cellular oxidative stress and anti-inflammatory actions.^135^^,^^136^ Thus, accumulating evidence indicates that ketamine decreases lipid peroxidation and protein damage while increasing the activity of antioxidant enzymes such as superoxide dismutase and catalase in the prefrontal cortex (PFC), hippocampus (HC), and nucleus accumbens of male and female rats exposed to MD.^56^^,^^118^ Moreover, ketamine has shown to moderate neuroinflammatory processes,^136^ known to contribute broadly to depression and other brain disorders.^137^ Thus, the administration of ketamine to MD male and female rats reversed increased myeloperoxidase activity compared with control animals, an enzyme known to decrease levels of the proinflammatory cytokines IL-6 and tumor necrosis factor *α* in the PFC and HC.^56^^,^^119^ Ketamine also regulates the integrity of the blood–brain barrier (BBB), which is disrupted by ELS.^138^ Of particular importance, ketamine restored BBB integrity in the PFC of male MD rats.^117^

Finally, there has been interest in minocycline, an inhibitor of microglia activation,^139^ which reduces glutamate neurotransmission and exhibits antidepressant properties.^140^ It is postulated that minocycline may exert its antidepressant effects through interactions with the midbrain dopamine (DA) systems. Thus, in 1 noteworthy recent study, early social stress caused a range of dopaminergic abnormalities in the ventral tegmental area of male but not in female mice, including changes in neuronal morphology and expression levels of the DA transporter and tyrosine hydroxylase.^120^ Remarkably, minocycline rescued these effects, suggesting a role for microglia in the effects of social stress on the developing DA systems.

## 9 Serotonergic interventions

The brain serotonergic systems are exquisitely sensitive to stress across the life span.^7^ Arguably, as the most complex of the ascending monoaminergic neurotransmitters systems, with more than 20 G protein–coupled receptors (GPRs), brain serotonin (or 5-HT) has been extensively associated with mood and emotional states and is an important substrate for therapeutic interventions in depression and anxiety.^141^^,^^142^ Moreover, as reviewed further, SSRIs and drugs that block 5-HT2 receptors have been consistently shown to prevent or reverse ELS-induced deficits in the negative valence and cognitive systems. Thus, chronic administration of SSRIs such as escitalopram, fluoxetine, and imipramine decreased immobility time on the FST in rats exposed either to MS^117^^,^^119^^,^^121^ or PS.^143^ Moreover, the combination of citalopram and the probiotic *Bifidobacterium infantis* reduced immobility and increased swim time in MS rats.^123^ These compounds have also been shown to rescue anhedonia-like behaviors measured by self-grooming behavior in the splash test, with the combined treatment of escitalopram, ketamine, and ECS increasing grooming time in male MD rats.^119^ However, the administration of paroxetine during late adolescence failed to prevent depression-like behaviors caused by LBN.^144^

Other compounds of interest include those with affinity for the 5-HT_2A_ receptor. For example, the 5-HT_2A_ receptor antagonist ketanserin blocked the emergence of anxiety-like behavior in adult rats exposed to MS,^124^ assessed using both the EPM and OFT. At a molecular level, ketanserin normalized the stress-induced impact on immediate early gene expression, particularly in the PFC.^124^ Ketanserin treatment also prevented MS-induced upregulation of specific genes associated with 5-HT2 receptor signaling, including Prkcb1, Plek, and Ppp3ca and 5-HT_2A_ mRNA levels.

Serotonergic drugs are also modulated by ELS in the context of the RDoC cognitive system domain. Thus, escitalopram administration in MS rats significantly increased the time spent in the target quadrant of the MWM.^121^ In contrast, subchronic treatment with desimipramine did not affect sustained attention and waiting impulsivity on the 5CSRTT in early socially isolated reared rats.^70^ However, rats reared in this way showed a blunted impulsivity response to *d*-amphetamine on the 5-CSRTT.^69^ These findings contrasted with motor hyperactivity in early socially isolated rats.

GPR55 has also been implicated in the mechanism underlying the effects of MS on the brain 5-HT systems. Thus, deficits in expression levels of GPR55 in MS mice were associated with impairments on NOR, Y-maze, and MWM.^145^ Intriguingly, selective activation of GPR55 with the agonist O-1602 increased tryptophan hydroxylase 2 protein and mRNA levels in dorsal raphe neurons, increased 5-HT release, and enhanced learning and memory in the NOR, Y-maze, and MWM tests.

## 10 Cannabinoid interventions

Cannabinoid (CB) receptor agonists and ECB-degrading enzymes are increasingly recognized to offset the deleterious effects of ELS on at least 3 RDoC systems: the negative valence system; cognitive valence system, and the social system. The ECB system is a neuromodulatory lipid-based system that includes a group of endogenous lipid mediators and neurotransmitters such as *N*-arachidonoylethanolamine (anandamide) and 2-arachidonoylglycerol.^125^ ECB signaling is controlled in part by selective ECB-degrading enzymes, such as fatty acid amide hydrolase (FAAH), which are responsible for the degradation of anandamide and monoacylglycerol lipase, involved in the degradation of 2-arachidonoylglycerol.

Administration of the cannabinoid receptor subtype 1/2 (CB1/2) agonist WIN55,212-2 during late adolescence (P45–60) prevented impairments in short-term memory caused by ELS, specifically as assessed in the OLT and social recognition (SR) tasks. This compound also reduced anxiety levels in the OFT in male and female rats exposed to LBM.^126^ However, administration of WIN55,212-2 during the early adolescent period (P30–P45) impaired spatial and SR tests in male rats exposed to LBM, suggesting the importance of neurodevelopmental timing effects. Indeed, treatment of male and female MD with the FAAH inhibitor URB597 or the monoacylglycerol lipase inhibitor JZL184 for 2 weeks during the late-adolescence period (P45–P60) prevented behavioral impairments on the social preference test and the PFC-dependent short-term SR memory test.^127^ In contrast, the administration of the CB1 antagonist AM251 alone had no significant effect on behavior in either adult males or females after MD stress. Nevertheless, AM251 attenuated the beneficial effects of URB597 and JZL184 on SR, immobility in the FST and anxiety in the OF test, thus highlighting the CB1 receptor in preventing the deleterious effects of ELS. This conclusion was replicated and extended by Portugalov et al^144^ in which administration of URB597 during late-adolescence (P45–P60) prevented the development of a depressive-like phenotype in ELS male and female rats exposed to LBN. In males, URB597 restored ELS-induced hypoactivity and freezing in the OFT and decreased SR and learned helplessness in the FST. In females, URB597 restored the ELS-induced decrease in SR as well as immobility in FST. Moreover, in this study, ELS downregulated specific microRNAs (miR-16 in males and miR-135a in females) in the PFC, which were restored by URB597 treatment. Again, highlighting the importance of sexual dimorphism in ELS effects, the increase in cnr2 and decrease in FAAH mRNAs in the PFC were reversed by URB597 treatment only in females.

## 11 Other pharmacological treatments

In recent years, alternative treatments have been proposed to attenuate the negative consequences of ELS on the social and positive valence systems. One of the most promising interventions is the use of the neuropeptide oxytocin (OT). OT plays a multifaceted role in the central nervous system, influencing both social and nonsocial behavior. However, the clinical application of OT is limited by its short half-life and poor ability to cross the BBB when administered systemically. Intranasal administration is thought to bypass the BBB, allowing OT to enter the brain directly. Specific interventions have shown that OT mitigates the effects of ELS induced by MS on the social system, cognitive system, and positive valence system. Thus, intranasal OT treatment from P22 to P34 in rats improved prosocial behavior in a T-maze, spatial learning and memory, and long-term potentiation after ELS induced by MS.^129^^,^^130^ Moreover, the combination with environmental enrichment (EE) had a positive impact on ELS-exposed rodents. In this procedure, EE involved a larger cage to provide more space for exploration, various enrichment objects (eg, toys, tunnels, running wheel, ladder, plastic blocks, swing, slide, and a nest), and greater opportunities for social encounters by increasing the number of rats in each cage. Both EE and intranasal OT administration effectively mitigated the decision-making deficits on rGT caused by MS. Thus, combined EE and OT significantly increased the percentage of advantageous choices compared with the MS-alone group indicative of an improvement in advantageous decision-making.^131^ Previous studies have demonstrated the beneficial effects of EE in reducing anxiety-like behavior in the EPM.^146^ These beneficial effects depended on the number of days rats were exposed to EE (ie, 10, 20, or 40 days) with the greatest improvements observed following 20- and 40-day exposures. Intriguingly, the authors demonstrated that the underlying neurobiological mechanisms of this EE benefit were related to a reduction in oxidative stress.

Other studies, cited earlier, have shown partial beneficial effects of probiotic treatment with *B infantis*.^117^ Probiotic treatment reversed the behavioral deficits observed in female rats exposed to MS on the FST. However, this effect was not observed in male rats, nor did this probiotic ameliorate anxiety-like grooming behavior in either female or male rats. The authors speculated that these effects may have been mediated by an increase in BBB integrity in female rats together with reduced oxidative stress in the PFC and the HC. Along similar lines, other research has investigated the effects of myosin light chain kinase, which regulates tight junction contraction and controls intestinal permeability.^147^ Myosin light chain kinase prevented the deleterious effects of MS on sexual reward seeking in male rats and reversed stress-induced changes in corticosterone levels during adulthood. This effect was accompanied by a normalization of the gut microbiome in MS rats.^47^

Finally, a recent study has revealed the possible therapeutic effect of paeoniflorin (PF), an anti-inflammatory substance derived from *Paeonia lactiflora*,^148^ to restore the deleterious effects of ELS in the RDoC motor, negative valence, and cognitive systems. Chronic administration of PF from P38 to P65 improved various behaviors in stressed rodents, including total distance traveled in the OFT, anxiety-like behaviors in the EPM, learning and memory in the MWM, and fear memory in the FC test.^133^ The precise mechanism of this effect is unknown but may involve the balance in activity between histone acetyltransferases and histone deacetylases, both key enzymes involved in histone acetylation and epigenetic modification.^149^

## 12 Reconciling the effects of ELS on centrally active pharmacological agents: an RDoC informed approach

Many studies reviewed in this article fall within the negative valence system and the cognitive system of the RDoC framework. Rather surprisingly, fewer studies have investigated the effects of ELS on the social processes system.

In the negative valence system effects, the various ELS studies reviewed show clear consistency in the loss construct, assessed on the FST by increased immobility time on the FST, decreased swimming, and reduced latencies to despair on the FST. Moreover, the NMDA receptor antagonist, ketamine robustly reversed these effects in rodents exposed to MD^48^^,^^118^^,^^119^ and MS.^117^ However, the effects of serotonergic agents have generally been more mixed, especially following chronic treatment regimens. For example, beneficial effects of escitalopram at 10 mg/kg^117^ and 25 mg/kg^121^ are reported in MS animals. Similar effects are reported for fluoxetine and imipramine^143^ after PS and citalopram combined with probiotics after MS.^123^ In contrast, chronic treatment with paroxetine (5 mg/kg) did not prevent the depressive phenotype on the FST in LBN animals.^144^ Finally, with reference to the loss construct, CBs also reverse immobility time on the FST following the chronic administration of URB597 and JZL184 in LBN animals.^127^^,^^144^

ELS causes consistent effects on the potential threat construct within the negative valence system. For example, anxiety-like behavior in the OFT is rescued by ketamine after MD stress,^56^ the 5-HT antagonist ketanserin after MS stress,^124^ the CB agonist WIN55-212 after LBN stress,^126^ and OT after MS stress.^129^ Moreover, grooming behavior in the splash test is reversed by ketamine administration in MD animals.^118^^,^^119^ Further, anxiety-like behavior on the EPM is reversed by the 5-HT antagonist ketanserin in MS animals,^124^ EE for 10, 20, and 40 days in MD animals,^146^ and PF after prolonged stress exposure.^133^ This later study also demonstrated that PF affected the acute threat construct, by reducing freezing behavior in the FCT.

With respect to cognitive system domain, a range of compounds have shown beneficial effects on spatial and nonspatial memory in rodents subjected to ELS. For example, spatial memory deficits of ELS animals in the MWM were rescued by the SSRI escitalopram,^131^ a GPR55 agonist,^145^ an ECB agonist,^126^ OT,^129^ and PF.^133^ Moreover, the assessment of nonspatial memory by NOR test revealed an enhanced performance of MS animals following administration of a GPR55 agonist.^145^ In relation to the cognitive and positive valence systems, recent results have shown that OT and EE improved decision making in the rGT by increasing advantageous choices in MS animals.^131^

## 13 Concluding remarks

This review provides a comprehensive survey of ongoing research on the neurobehavioral consequences of ELS considered within the RDoC framework. ELS leads to persistent abnormalities in the emotional and cognitive domains, which can be remediated by selective pharmacological agents targeting the glutamatergic, serotonergic, and ECB systems. It is noteworthy that interventions targeting the oxidative stress and neuroinflammatory pathways are particularly efficacious in reversing the long-term consequences of ELS on the negative valence and cognitive systems, including ketamine, SSRIs, CBs, and OT. More generally, our findings highlight ELS as a critical modulator of drug action in the central nervous system. By reverse engineering the mode of action of clinically effective drugs for depression and other stress-related disorders, it is anticipated that a much richer understanding of the neurobehavioral consequences of ELS will be forthcoming.

## Conflict of interest

The authors declare no conflicts of interest.
